# Supplementary material for: Model predictive path integral for decentralized multi-agent collision avoidance
Source: PeerJ Comput Sci. 2024 Aug 21;10:e2220. doi: 10.7717/peerj-cs.2220 (PMC11639165; doi:10.7717/peerj-cs.2220)
Supplement: Supplemental Information 1 [file peerj-cs-10-2220-s001.pdf]

APPENDIX

A PSEUDOCODE FOR MODEL PREDICTIVE PATH INTEGRAL FOR MULTI-AGENT COLLISION AVOIDANCE

Algorithm 1 MPPI FOR MULTI-AGENT COLLISION AVOIDANCE

Input:
K – number of sampled trajectories; T – planning time horizon; i – current agent; $\mathbf{x_0}$ – current agent state;
$\mathcal{N}_i$ – neighboring agents; $X_{\mathcal{N}_i} = \{(p_x^j, p_y^j, v_x^j, v_y^j, r^j) \mid j \in \mathcal{N}_i\}$ – information on neighboring agents;
$r(\cdot)$, $\phi(\cdot)$, $c'(\cdot)$ – running, terminal and control cost functions; $F(\cdot)$, $G(\cdot)$ – transition functions;
$\mathbf{u_{min}}$, $\mathbf{u_{max}}$ – control limits; α – desired safety probability; λ – MPPI hyperparameter;
$\mathbf{u^{init}}$ – default initial control action; $U^{init} = \{\mathbf{u_0^{init}}, ..., \mathbf{u_{T-1}^{init}}\}$ – Initial control sequence (obtained from previous step)

Predict neighbors trajectories $\pi_1, \pi_2, ..., \pi_N$
Compute linear constraints $ORCA^{\tau} = \{ORCA^{\tau}_{i|j} \mid j \in \mathcal{N}_i\}$ based on $X_{\mathcal{N}_i}$
Find μ', Σ' based on $\mathbf{u_0^{init}}, \Sigma$, α and $ORCA^{\tau}$
for $k = 1$ to K **do**
    Sample $\varepsilon_{\mathbf{0}}^{\mathbf{k}} \sim \mathcal{N}(\mu', \Sigma')$
    Sample $\{\varepsilon_{\mathbf{1}}^{\mathbf{k}}, ..., \varepsilon_{\mathbf{T}}^{\mathbf{k}}\}, \varepsilon_{\mathbf{t}}^{\mathbf{k}} \sim \mathcal{N}(0, \Sigma)$
    for $t = 1$ to T **do**
        $\mathbf{u_{t-1}^k} \leftarrow \mathbf{u_{t-1}^{init}} + \varepsilon_{\mathbf{t-1}}^{\mathbf{k}}$
        Limit $\mathbf{u_{t-1}^k}$ using bounds $\mathbf{u_{min}}$ and $\mathbf{u_{max}}$
        $\mathbf{x_t} \leftarrow F(\mathbf{x_{t-1}}) + G(\mathbf{x_{t-1}})\mathbf{u_{t-1}}$
        $S(\tilde{U}^k) \leftarrow S(\tilde{U}^k) + r(\mathbf{x_t}) + c'(\mathbf{u_t}))$
    end for
end for
$S(\tilde{U}^k) \leftarrow S(\tilde{U}^k) + \phi(\mathbf{x_T})$
$\rho \leftarrow \min_k S(U_k)$
$\eta \leftarrow \sum_{k=1}^{K} \left(exp\left(-\frac{1}{\lambda}\left(\tilde{S}(U^k) - \rho\right)\right)\right)$
for $k = 1$ to K **do**
    $\omega(U^k) \leftarrow exp\left(-\frac{1}{\lambda}\left(\tilde{S}(U^k) - \rho\right)\right)$
end for
for $t = 0$ to $T - 1$ **do**
    $\mathbf{u_t^*} = \sum_{k=1}^{K} \left(\omega(U^k)\mathbf{u_t^k}\right)$
end for
$\mathbf{u_{result}} \leftarrow \mathbf{u_0^*}$
for $t = 1$ to $T - 1$ **do**
    $\mathbf{u_{t-1}^{init}} \leftarrow \mathbf{u_t^*}$
end for
$\mathbf{u_{T-1}^{init}} \leftarrow \mathbf{u^{init}}$
return $\mathbf{u_{result}}$ and new control sequence U^{init} for next step

B OPTIMIZATION PROBLEM FORMULATION FOR SINGLE-INTEGRATOR DYNAMICS

In case of single-integrator dynamics case, the control vector is defined as velocity of agent $\mathbf{u_t} = (v_{x,t}, v_{y,t})$ and state defined as position $\mathbf{x_t} = (p_{x,t}, p_{y,t})$. So, the transition functions and equation of movement are the following:

$$F(\mathbf{x_t}) = \mathbf{x_t};\ G(\mathbf{x_t}) = \begin{pmatrix} 1 & 0 \\ 0 & 1 \end{pmatrix} \tag{36}$$

$$\begin{pmatrix} p_{x,t+1} \\ p_{y,t+1} \end{pmatrix} = \begin{pmatrix} p_{x,t} \\ p_{y,t} \end{pmatrix} + \begin{pmatrix} 1 & 0 \\ 0 & 1 \end{pmatrix} \begin{pmatrix} v_{x,t} \\ v_{y,t} \end{pmatrix} \tag{37}$$

Let $\mu_{v_x}, \mu_{v_y}, \sigma_{v_x}, \sigma_{v_y}$ be initial distribution parameters and $\mu'_{v_x}, \mu'_{v_y}, \sigma'_{v_x}, \sigma'_{v_y}$ be new parameters. We also involve an auxiliary variable $t_{\mu'_{v_x}}, t_{\mu'_{v_y}}, t_{\sigma'_{v_x}}, t_{\sigma'_{v_y}}$ to replace the module in objective function with linear expression. Thus, elements of SOCP linear objective function can be defined in next form:

$$\begin{aligned} &\mathbf{f} = (0,0,0,0,1,1,1,1), \\ &\mathbf{x} = (\mu'_{v_x}, \mu'_{v_y}, \sigma'_{v_x}, \sigma'_{v_y}, t_{\mu'_{v_x}}, t_{\mu'_{v_y}}, t_{\sigma'_{v_x}}, t_{\sigma'_{v_y}}) \end{aligned} \tag{38}$$

Then, the objective function and linear constraints are the following:

$$\begin{aligned} \text{minimize} \quad & t_{\mu'_{v_x}} + t_{\mu'_{v_y}} + t_{\sigma'_{v_x}} + t_{\sigma'_{v_y}} \\ \text{s.t.} \quad & -t_{\mu'_{v_x}} \le \mu'_{v_x} - \mu_{v_x} \le t_{\mu'_{v_x}} \\ & -t_{\mu'_{v_y}} \le \mu'_{v_y} - \mu_{v_y} \le t_{\mu'_{v_y}} \\ & -t_{\sigma'_{v_x}} \le \sigma'_{v_x} - \sigma_{v_x} \le t_{\sigma'_{v_x}} \\ & -t_{\sigma'_{v_y}} \le \sigma'_{v_y} - \sigma_{v_y} \le t_{\sigma'_{v_y}} \\ & \mu'_{v_x} + \Phi^{-1}(\alpha)\sigma'_{v_x} \ \le \ v_{x,max}, \\ & \mu'_{v_x} - \Phi^{-1}(\alpha)\sigma'_{v_x} \ \ge \ v_{x,min}, \\ & \mu'_{v_y} + \Phi^{-1}(\alpha)\sigma'_{v_y} \ \le \ v_{y,max}, \\ & \mu'_{v_y} - \Phi^{-1}(\alpha)\sigma'_{v_y} \ \ge \ v_{y,min}, \\ & \sigma'_{v_x} \ \ge 0,\ \sigma'_{v_y} \ \ge 0, \end{aligned} \tag{39}$$

Let a_j, b_j, c_j be coefficients of ORCA linear constraint for some neighbor j. Then, inequality from Eq. 30 parameters can be written in next form:

$$A'_j = \begin{pmatrix} 0 & 0 & a_j & 0 & 0 & 0 & 0 & 0 \\ 0 & 0 & 0 & b_j & 0 & 0 & 0 & 0 \end{pmatrix}, \tag{40}$$

$$b'_j = -c_j \tag{41}$$

$$\mathbf{c'_j} = -\frac{1}{\Phi^{-1}(\alpha)} \cdot (a_j, b_j, 0,0,0,0,0,0) \tag{42}$$

C OPTIMIZATION PROBLEM FORMULATION FOR DIFFERENTIAL-DRIVE ROBOT DYNAMICS

In case of differential-drive robot dynamics, described in Eq. 3, the optimization problem can be represented as linear program (LP).

First, let's repeat the steps described in section *"Finding Safe Distribution Parameters"* and shown in appendix B on an example of single-integrator dynamics. Let $\mu_v, \mu_w, \sigma_v, \sigma_w$ be initial distribution parameters and $\mu'_v, \mu'_w, \sigma'_v, \sigma'_w$ be new parameters. We also involve an auxiliary variable $t_{\mu'_v}, t_{\mu'_w}, t_{\sigma'_v}, t_{\sigma'_w}$ to replace module in objective function with linear expression. Thus, elements of SOCP linear objective function can be defined in next form:

$$\begin{aligned} &\mathbf{f} = (0,0,0,0,1,1,1,1), \\ &\mathbf{x} = (\mu'_v, \mu'_w, \sigma'_v, \sigma'_w, t_{\mu'_v}, t_{\mu'_w}, t_{\sigma'_v}, t_{\sigma'_w}) \end{aligned} \tag{43}$$

Then, the objective function and linear constraints are the following:

$$\begin{aligned} \text{minimize} \quad & t_{\mu'_v} + t_{\mu'_w} + t_{\sigma'_v} + t_{\sigma'_w} \\ \text{s.t.} \quad & -t_{\mu'_v} \le \mu'_v - \mu_v \le t_{\mu'_v} \\ & -t_{\mu'_w} \le \mu'_w - \mu_w \le t_{\mu'_w} \\ & -t_{\sigma'_v} \le \sigma'_v - \sigma_v \le t_{\sigma'_v} \\ & -t_{\sigma'_w} \le \sigma'_w - \sigma_w \le t_{\sigma'_w} \\ & \mu'_v + \Phi^{-1}(\alpha)\sigma'_v \ \le \ v_{max}, \\ & \mu'_v - \Phi^{-1}(\alpha)\sigma'_v \ \ge \ v_{min}, \\ & \mu'_w + \Phi^{-1}(\alpha)\sigma'_w \ \le \ w_{max}, \\ & \mu'_w - \Phi^{-1}(\alpha)\sigma'_w \ \ge \ w_{min}, \\ & \sigma'_v \ \ge 0, \ \sigma'_w \ \ge 0, \end{aligned} \tag{44}$$

Next, we will find inequality (Eq. 30) parameters based on ORCA linear constraint coefficients a_j, b_j, c_j for some neighbor j. Then, inequality from Eq. 30 parameters can be written in next form:

$$A'_j = \begin{pmatrix} 0 & 0 & [a_j \cos\theta_t + b_j \sin\theta_t] & 0 & 0 & 0 & 0 & 0 \\ 0 & 0 & 0 & 0 & 0 & 0 & 0 & 0 \end{pmatrix}, \tag{45}$$

$$b'_j = -c_j \tag{46}$$

$$\mathbf{c'_j} = -\frac{1}{\Phi^{-1}(\alpha)} \cdot ([a_j \cos\theta_t + b_j \sin\theta_t], 0,0,0,0,0,0,0) \tag{47}$$

It is easy to see, that final inequality can be represented as linear and not include any variable associated with w.

$$[a_j \cos\theta_t + b_j \sin\theta_t]\ \sigma_v \ \le \ \frac{[a_j \cos\theta_t + b_j \sin\theta_t]}{\Phi^{-1}(\alpha)} \cdot \mu_v \ - \ \frac{c_j}{\Phi^{-1}(\alpha)}, \tag{48}$$

So, we can exclude every variable associated with w from the problem 44, since these variables do not affect the consideration of collision avoidance constraints. Now, the final LP is obtained:

$$\begin{aligned} \text{minimize} \quad & t_{\mu'_v} + t_{\sigma'_v} \\ \text{s.t.} \quad & -t_{\mu'_v} \le \mu'_v - \mu_v \le t_{\mu'_v} \\ & -t_{\sigma'_v} \le \sigma'_v - \sigma_v \le t_{\sigma'_v} \\ & \mu'_v + \Phi^{-1}(\alpha)\sigma'_v \ \le \ v_{max}, \\ & \mu'_v - \Phi^{-1}(\alpha)\sigma'_v \ \ge \ v_{min}, \\ & \sigma'_v \ \ge 0, \\ & [a_j \cos\theta_t + b_j \sin\theta_t]\ \sigma_v \ \le \ \frac{[a_j \cos\theta_t + b_j \sin\theta_t]}{\Phi^{-1}(\alpha)} \cdot \mu_v \ - \ \frac{c_j}{\Phi^{-1}(\alpha)}, \ \forall j \in \mathcal{N}_i \end{aligned} \tag{49}$$
